# The Logic of the Situation—The New Pharmacy and the Old

**Published:** 1907-03

**Authors:** Woodbridge Hall Birchmore

**Affiliations:** Brooklyn, N. Y.; 163 Fulton Street


### The Logic of the Situation—The New Pharmacy and the Old.

By WOODBRIDGE HALL BIRCHMORE, M. D., Brooklyn, N. Y.

IT is now some decades, if not scores, of years since the step was taken, and taken by one of the great and highly respected manufacturers of pharmaceutical preparations, which recognised that in buying and selling crude drugs, and manufactured preparations also, the only just criterion of their worth must be their "alkaloidal strength", as it is called, "the strength as determined by the amount of the active ingredient contained," as a circular letter from this firm expressed the idea. This action was at the time a new departure in the trade and it had, as perhaps those who adopted this course intended, the effect of putting on one side, the goods of this house and on the other the articles offered to the trade by all the rest of the manufacturing chemist firms. The step was an advance, and it was a long one; by it was opened the era of the titred or assayed tinctures, an era of brief duration comparatively but of great importance for it was by the evidence of superiority of method, thus afforded, that the way was paved for the introduction to the medical profession of the alkaloids and alkaloidal salts as legitimate merchandise. It is interesting to consider somewhat in detail the events as they came in sequence, one after another, and to consider some of the important ones, both as conditioned by the past incidents and as conditioning those which followed. Doing this we are really discussing the forces which conditioned the development of a new materia medica, a materia medica which is proved to be so unlike the old that new theories are required to explain the mysterious problems, in respect to their therapeutic action, which clinical experience provides.

To discuss these questions properly, we must start from a date chronologically not distant, yet in respect to the development of therapeutic art, very long ago. In pharmacy the long period antecedent to this present one may be called the dark ages, and the action of drugs was at this time considered by most physicians to be as mysterious as the causation of disease. Theories of the actions of drugs existed as theories of the causation of disease existed, and these theories in a sense were complements of each other, but the idea that in drugs mysterious substances were to be found, and that these gave to the drugs their "potency" and usefulness, was already discussed and recognised as a logical necessity; and in spite of the fashionable theory that the true explanation of the usefulness of things was discovered by accident, the student entering this field with unbiased mind and pursuing investigation honestly soon sees that the discovery of the alkaloids was as distinctly the result of research guided by logical plans, as was the synthesis of urea from inorganic substances by Wohler.

The question of priority in respect to this discovery of the alkaloids has been discussed from special points of view by a number of specialists, but with the claims of the chemists in regard to this alkaloid or that one we have nothing to do for at the time which we have in mind for consideration, the alkaloids had not yet been seen except in the dreams of certain men. Crude drugs were purchased by men depending upon the personal skill and judgment to aid them in making choice of the proper article. Opium is valued by these buyers, not by the amount of morphine it contains, but according to the opinion of the purchasing "chemist" or dealer, as are all the plants known as simples. Scientific botany is scarce born, but practical botany, as the art of distinguishing species is already pretty well advanced. The names quinine, cinchonine, chinconidine are not yet imagined possibilities, but the physical distinctions between the various "Peru barks" are already known. Four kinds at least are considered and we read in books of the day of "red," "yellow," "Quito," and "Amazons" bark. That the distinction in the activities of the different species must be caused by difference in the "subtle essence contained therein" none question, but how the essences differ none can say. What these, "subtle essences" may be, many speculate, none know, but that they can be removed from the drug by grinding it fine and extracting the powder with "spirits of wine," that is by using as solvent more or less dilute alcohol, is well, very well, known and has been known from time out of mind.

When governments, for the sake of revenue, not to suppress the trade, began to tax the "distilled spirits of wine" and do so according to its tested strength, a new element is introduced. Historians note how the test is made, but it is as refined as the conditions demand. The relation of strength to specific gravity is next recognised, and the strength of the pharmaceutical spirit is "standardised" so to say. Other improvements are made and after a time some one distils the spirits of wine with "potash," and decides that this is the proper way to do the thing, defines the technique, and then the technique for the manufacture of the tinctures is standardised again with "alcohol" instead of the "spirit of wine."

The menstruum was thus determined and the problem was this much at least simplified, but if pleased with the good fortune in respect to the menstruum the physician, the apothecary, was as much at a loss as ever in respect to the drug which he used. The best he could do was to purchase the very highest grade of raw material and so far as he could determine it, but be as careful as he might, considered medicinally his preparations would vary, and vary much, quite too much in their strength and consequent action upon the patient. That this unforseeable variation in strength of the various pharmaceutical preparations is the *radix funesta malorum* which gave occasion for the perfectly well-justified hesitation of the physicians in administering remedies none can doubt, and while it hindered the free action of the physician in one direction, it confined his action in all. Did a new sample of opium come to hand, its strength could not be determined almost instantly by assaying the contents for the total alkaloidal strength and total morphine, but the user must carefully make trial of its effects, as if it were a new drug just introduced to professional notice, as indeed it might be considered to be.

Such was the condition of things when the true meaning of the contradictory actions of one special drug—namely, opium, was made clear, or at least was rendered less incomprehensible.

During about a quarter of a century, or such a matter, evidence that substances having definite chemical reactions could be obtained from drugs had been accumulating. But that the action of the drug was the sum of the reactions of a number of constituent alkaloids, and could be expressed in mathematical language, did not come into view until some time afterwards. The ground therefore was ready and the seed was sown, but before the crop could be gathered the plants must grow.

It would be interesting indeed, did space permit, to tell in detail the development from information into knowledge concerning "The Cinchona and its Alkaloids," but the elaborate study under the above title is so easily to be obtained and the book is so admirably written that the obvious course is to refer the reader to it. This fact may be mentioned, that at once the proper method of manufacture was shown to be practical, its commercial development was quite assured, and the story of the development of the secondary alkaloids is essentially part of the history of quinine, of its salts and of the chemists who made it and whom it made.

In respect to the opium alkaloids the tale is by no means so commonplace. For ages it had been known to the physicians that opium usually brought sleep, but any given sample might cause vigil; that almost always it dulled the sense of pain, but it might excite the sense; almost always it quieted spasm, but it had been known to cause convulsion. But when it was discovered that while the morphine content varied greatly, as did the total alkaloids, but not always in the same direction, a clue was at once suggested by which in course of time the puzzling complication has been unwound. In a word, opium contains many alkaloids, glucosides and two acids, and every individual in this complicated group may cause its own reaction variously modifying all the rest.

From the very instant a process of separation into the components was devised, the manufacture of the cinchona alkaloid quinine was begun, and as soon as morphine could be made with certainty, this was the product for which many labored patiently.

The business of the great chemical manufactories began at once, but it did not interfere with the work of the pharmacist on the corner; the business which began to apply the laws of naturel selection here was that of the great manufacturing pharmacists, who were beginning to introduce their lines of assayed extracts and such like. But almost at once these houses saw that by an alliance with the dispensing pharmacist he could be taught to buy not crude drugs but, "concentrated extracts," and by diluting these concentrates with so much alcohol, the U. S. P. fluid extract, or the U. S. P. tinctures could be made. So much did this system of "assayed standard concentrates" benefit the retailer that he was able to use this line of goods in filling prescriptions and to sell the standard quality at prices which would not have paid for the raw materials had he sought to "maintain his independence," by continuing to make the tinctures from the official raw materials as these are defined in the pharmacopeia and by its methods.

While the manufacturing pharmacists gave this reason to the dispenser for using their preparations diluted as by directions just noted instead of making the tinctures by percolation from alcohol and the raw drug, they gave quite different reasons to the physicians as an inducement to insist upon the use of assayed preparations, even this one, that these tinctures *always contained* the same amount of the *alkaloids* of the drug in any given dose. That every fluid ounce of the fluid extract contained the same as every other fluid ounce no matter when purchased, because both were *assayed* for their *alkaloidal* strength and were made up to the same titre.

It really does seem a little strange that this circular did not suggest to the physicians and to the chemists (but perhaps it did) and to the drug store man on the corner (perhaps to him also the suggestion came) that in actual fact the value was in the alkaloid, or other proximate principles contained, and not to the miscellaneous impurities of one kind and another associated with the alkaloid, and that the part of wisdom would be to use the alkaloid, and the alkaloid alone as materia medica, yet the suggestion does not appear anywhere.

But meanwhile the chemists were steadily as work determining the active principles, the alkaloids and glucosides present in the drugs and as carefully isolating them so that almost before any one really understood what was happening, a new materia medica was at the disposal of the physicians. Chemist after chemist has added here one and then another to the list until the active chemical entity in nearly every medicinal plant has been determined and isolated. The meaning of all this work from the physicians' view point is presently to be seen and certain manufacturers begin to put the new materia medica into a form in which it can be made useful in practice. The possibilities begin to be the subject of discussion, and for every dose of medicine prescribed, twenty words are used. In this direction the pharmacist sees danger, in that the manufacturing chemist, hence many excited men voiced their views of the situation with tongue and discussed it with pen.

But while much is written and more is said, the volume of words spoken and written comes practically from one group only, because it appears to not a few, that there is no room for argument. The reason for using the chemists' preparations rather than the pharmacists', appears to be obvious to many, but to a few the idea of the change is distasteful, very; they see in it an unwelcome revolution. But while the chemists are simply preparing for war, the champions of the pharmacists are already beginning to tell the reasons why they are the men who have the right to be and to do and not these "makers of novelties." As if the chemists were in any sense making anything! They are but parting one from another those wonderful compounds which the plants have made, and are giving them singly to the physicians while the pharmacist hands them out all at once in a mixed handful. The pharmacist forgets the assayed tincture which he boasts of using, tells us that it contains all the medicinal qualities of the plant just as nature made (and mixed) them, not artfully parted by the hand of man. Granted that this is true, what then? Does this show that powdered gum opium is to be given in preference to morphia sulphate? By no means, we think. The pharmacist's side is very easily set forth; but he has prejudged his cause long since, when he welcomed the assayed extracts, for by welcoming them he acknowledged the importance of the alkaloid and glucoside, and now he can not avoid the difficulty which his previous acknowledgment has made for him. So now he is up against it, and badly. The alkaloid, the alkaloidal salts, the glucoside is at the hand of the physician, hence this campaign.

The question is easily put by the physician, "You ask me to continue to use the preparations which you make and to cease to use the preparations which come from the chemist.

Now answer me these questions:

1. Is it not fact that the amounts and proportions of the active ingredients in any plant are variable quantities?

2. Is it not a fact that the proportions vary much more relatively than the gross sum of all?

3. Does it now then follow that in making some one ingredient come to the titre you may, and in practice do, add proportions of other ingredients which you do not consider, but which for all you know may be very active?

4. Is it not a fact that in almost every known medicinal plant there are substances utterly useless as medicine, but which make the contents bulk needlesesly?

5. Would you consider that a physician was doing his duty if he went to your prescription case and made up powders without using the scales and without noting the contents of the bottles? Or a potion in the same way?

6. Is not the giving of such a preparation as laudanum an exact case in point, since laudanum contains more than a dozen ingredients which are never twice in the same proportion in any two opium balls and of which the morphine may be anything from five per cent. to twenty-two, the other ingredients varying inversely?

7. Is it not a fact that the hyosciamus niger and albus have been used more or less in medicine from the very beginning, and that because of the uncertainty of its action the drug has always been counted dangerous?

8. Is it not a fact that the two alkaloids hyoscyamine and hyoscine have been proved to be perfectly safe and of the most extreme usefulness, the unwished-for complications having been caused wholly by contamination with the great variable content of atropine?

9. Is it then not a manifest fact that *if we can* part the active ingredients in any plant from the woody fibre and such like, we ought to do so, hence tinctures; and if we can part the active ingredients from the useless ones and each other, we should do so for the same reason, hence alkaloids?

10. Since the alkaloid is an unvarying substance, and of unvarying action, does it not follow that it is more suitable for medicinal use than a substance about which we know nothing except that it is never twice the same?

11. Is it not a fact that the physician can by using the alkaloidal salts administer a medicine whose action he can predicate as exactly as anything can be predicated with respect to living tissue-reaction?

12. Now since you have answered every one of these questions in the affirmative, please tell me why I should use *anything other than alkaloids* as drugs?"

I can understand that when the substitution of the tiny pellet containing the dose of the alkaloid for the galenical preparations is made everywhere, "the drugstore on the corner" must cease to be the place whence medicines are issued to the patient,—but there are few drug stores which would seriously miss their prescription trade if they lost it,—at least so very many say, and plainly the use of the alkaloids is proved to be the proper course by "The Logic of the Situation."

163 Fulton Street.

---

Diabetes Mellitus.—Tyson advocates oxidising agents, such as arsenic, iron and potassium chlorate, with bathing, massage and outdoor exercises.—*Denver Med. Times.*

